# The Importance of High‐Density Microelectrode Arrays for Recording Multi‐Scale Extracellular Potential and Label‐Free Characterization of Network Dynamics in iPSC‐Derived Neurons

**DOI:** 10.1002/alz70855_099682

**Published:** 2025-12-23

**Authors:** Karin Morandell, Laura D'Ignazio, Elvira Guella, Silke Seyock, Praveena Manogaran, Silvia Ronchi, Marie Engelene J. Obien

**Affiliations:** ^1^ MaxWell Biosystems, Zurich, Zurich, Switzerland

## Abstract

**Background:**

Advances in microelectrode array (MEA) technology have enabled researchers to study neuronal networks across multiple scales, from subcellular properties to network‐level dynamics. These devices are critical for understanding the phenotypes of neurological disorders and advancing drug discovery, as they provide unique insights into neuronal network behavior. Key factors such as electrode density, spacing, and size significantly impact signal quality, noise, and sensitivity.

**Method:**

To exhaustively characterize neuronal networks, we utilized the MaxOne and MaxTwo high‐density (HD) MEA systems (MaxWell Biosystems, Switzerland) to record activity from induced pluripotent stem cell‐derived neurons. These systems, with 26,400 electrodes per well, enabled high‐resolution data collection over time. HD‐MEA recordings were compared to simulated low‐density recordings, where adjacent HD‐MEA electrodes were clustered to mimic larger, low‐density electrodes. The AxonTracking Assay, an automated tool for analyzing axonal structures simultaneously, was also used to assess axonal arbours and network functionality.

**Result:**

Higher electrode density and smaller electrode size enhanced sensitivity, allowing the detection of smaller spikes and capturing the full spectrum of network dynamics. The high‐density systems provided increased statistical power for longitudinal studies, while the AxonTracking Assay delivered insights into axonal structures and network activity.

**Conclusion:**

The combination of high‐resolution HD‐MEA recordings and advanced subcellular analysis tools offers a robust platform for drug screening and disease modeling, enabling researchers to better understand and target neuronal network dynamics in neurological disorders.

